# Extracellular vesicles from obese and diabetic mouse plasma alter C2C12 myotube glucose uptake and gene expression

**DOI:** 10.14814/phy2.15898

**Published:** 2024-01-02

**Authors:** Christopher R. Pitzer, Hector G. Paez, Peter J. Ferrandi, Junaith S. Mohamed, Stephen E. Alway

**Affiliations:** ^1^ Center for Muscle, Metabolism and Neuropathology, Division of Regenerative and Rehabilitation Sciences, College of Health Professions The University of Tennessee Health Science Center Memphis Tennessee USA; ^2^ Department of Physiology, College of Medicine The University of Tennessee Health Science Center Memphis Tennessee USA; ^3^ Integrated Biomedical Sciences Graduate Program, College of Graduate Health Sciences The University of Tennessee Health Science Center Memphis Tennessee USA; ^4^ Laboratory of Muscle Biology and Sarcopenia, Department of Physical Therapy, College of Health Professions The University of Tennessee Health Science Center Memphis Tennessee USA; ^5^ Laboratory of Muscle and Nerve, Department of Diagnostic and Health Sciences, College of Health Professions The University of Tennessee Health Science Center Memphis Tennessee USA; ^6^ Tennessee Institute of Regenerative Medicine The University of Tennessee Health Science Center Memphis Tennessee USA

**Keywords:** extracellular vesicles, metabolism, muscle, obesity, type 2 diabetes

## Abstract

Recent studies have indicated a role for circulating extracellular vesicles (EVs) in the pathogenesis of multiple diseases. However, most in vitro studies have used variable and arbitrary doses of EVs rather than interpreting EVs as an existing component of standard skeletal muscle cell culture media. The current study provides an initial investigation into the effects of circulating EVs on the metabolic phenotype of C2C12 myotubes by replacing EVs from fetal bovine serum with circulating EVs from control mice or mice with obesity and type 2 diabetes (OT2D). We report that EVs associated with OT2D decrease 2‐NBDG uptake (a proxy measure of glucose uptake) in the insulin‐stimulated state compared to controls. OT2D associated EV treatment also significantly decreased myosin heavy chain type 1 (MHCI) mRNA abundance in myotubes but had no effect on mRNA expression of any other myosin heavy chain isoforms. OT2D‐associated circulating EVs also significantly increased lipid accumulation within myotubes without altering the expression of a selection of genes important for lipid entry, synthesis, or catabolism. The data indicate that, in a severely diabetic state, circulating EVs may contribute to insulin resistance and alter gene expression in myotubes in a manner consistent with the skeletal muscle phenotype observed in OT2D.

## 1 INTRODUCTION

Type 2 diabetes concomitant with obesity (OT2D) is a chronic metabolic disorder with a growing economic and healthcare burden (Bommer et al., 2018). OT2D is characterized by elevated blood glucose due to defective insulin signaling. Skeletal muscle, adipose tissue, and the liver are the primary insulin‐sensitive tissues, and insulin resistance results in metabolic dysfunction in all three of these organs, with each uniquely contributing to OT2D. Skeletal muscle is the largest and most metabolically active of these tissues, and it is responsible for the bulk of insulin‐stimulated glucose uptake and oxidation (Meyer et al., 2002). Insulin binding to its extracellular receptor results in an intracellular phosphorylation cascade that includes phosphorylation of AKT2 and results in a sharp increase in glucose uptake by the muscle (Garofalo et al., 2003). Glucose transporter protein‐4 (GLUT4) is the primary glucose transporter associated with OT2D because its translocation is a canonical event in the insulin signaling cascade downstream of AKT2 in skeletal muscle. Reduced function of other glucose transporter family proteins that are expressed in skeletal muscle may also contribute to the OT2D phenotype. These functions include acting as a secondary insulin‐responsive glucose transporter (GLUT12) (Stuart et al., 2009), basal glucose uptake (GLUT1) (Gaster et al., 2000; Richardson & Pessin, 2018), and mechanical load‐induced glucose transport (GLUT10) (Mcmillin et al., 2017). Preserving muscle metabolic health is a prime therapeutic strategy for OT2D because preserving glucose uptake and oxidation capacity reduces systemic hyperglycemic stress.

Prior research has identified marked skeletal muscle phenotype shifts in OT2D. These changes include a decrease in myosin heavy chain type 1 (MHCI) (Olsson et al., 2011), a stark increase in lipid oxidation with dampened glycolytic capacity and inability to shift between which substrate is utilized (termed metabolic inflexibility), and ectopic lipid accumulation within the muscle (Kelley & Mandarino, 2000). While it is unclear what causes the OT2D‐associated muscle fiber type shift, myocyte enhancer factor 2C (MEF2C) is known to regulate MHC gene expression to promote an oxidative phenotype in skeletal muscle (Potthoff et al., 2007). Mitochondrial membrane fractionation is also prominent in skeletal muscle in OT2D (Gundersen et al., 2020). Mitochondrial fission proteins such as DRP1 have been shown to play a role in mitochondrial network fractionation, resulting in increased production of reactive oxygen species and potentially exacerbating insulin resistance (Kugler et al., 2021; Lin et al., 2018), although it is not clear if this is a cause or an effect of OT2D. Capillary rarefaction and decreased angiogenic signaling are also known to occur in OT2D (Groen et al., 2014). This is consistent with an increase in more typically glycolytic MHC expression but is mismatched with the known dependence on lipids as a metabolic substrate in OT2D. While a potential defective generation of angiogenic signals by EVs after exercise in obesity has been reported (Weiss et al., 2022), it is unknown if circulating EVs alter angiogenesis in OT2D at baseline. There is also evidence of reduced muscle regeneration in OT2D, which may be due to a chronic increase in inflammatory mediators. One such mediator, osteopontin, serves to recruit macrophages to damaged areas and begin the process of regeneration by promoting inflammatory degradation of damaged tissue; this inflammation also triggers the activation of muscle stem cells (Motoyoshi & Kamakura, 2003). Intramuscular lipid accumulation in OT2D has also been previously shown to correlate to insulin resistance (Goodpaster et al., 2000). Regulation of skeletal muscle metabolism is complex and multifactorial; however, the role of extracellular vesicles (EVs) in muscle metabolism in OT2D is largely unknown.

EVs are secreted by almost all mammalian cells. EVs are newly appreciated as an important means of intracellular communication by acting as a reporter of the homeostasis of the secreting cell (Stahl & Raposo, 2019). EV cargo is altered in response to a multitude of stimuli and is released into the interstitial space (Chen et al., 2021). EVs may act as extracellular signaling molecules (Al Halawani et al., 2022), or encapsulated EV cargo may enter recipient cells and alter their signaling. The discovery of this mechanism has led to an interest in EVs as disease biomarkers (Ludwig et al., 2021; Yoshioka et al., 2013), therapeutics (Claridge et al., 2021), and as potentially novel contributors to disease mechanisms (Wang et al., 2017). There are two primary populations of EVs that skeletal muscle cells are exposed to. This includes EVs coming from the circulation and those coming from neighboring muscle cells through the interstitial space. Both populations of EVs are important for muscle cell function, with circulating EVs impacting muscle cell homeostasis, especially in disease states (Miao et al., 2021), and muscle‐derived EVs serving important roles in maintaining satellite cell niche and regulating muscle stem cell proliferation and differentiation (Forterre et al., 2014; Hanson et al., 2023) and muscle regeneration (Suk et al., 2016).

There is an emerging body of literature describing the potential roles of circulating EVs in OT2D. Ying et al. report that exosomes (a highly bioactive class of EVs) from macrophages within the adipose tissue of obese mice impair insulin sensitivity in lean mice. This study also found that exosomes from adipose tissue in lean mice enhanced insulin sensitivity in their obese counterparts (Ying et al., 2017). This agrees with earlier findings from Deng et al., who showed that exosomes associated with obesity activate macrophages and induce insulin resistance (Deng et al., 2009). The contribution of adipose tissue macrophage‐derived EVs to OT2D phenotypes in skeletal muscle remains unclear; however, it is likely that these EVs are delivered to the muscle via the circulation.

The study of EVs presents interesting challenges, notably the contextual evaluation of the effects of cargo. A common approach for determining the role of EVs in disease contexts is to characterize cargo and then evaluate the effects of those cargo components independently through overexpression or exogenous treatment of cargo molecules (Di et al., 2021; Katayama et al., 2019; Wang et al., 2017). This approach is experimentally sound but does not allow precise modeling of the “dose” of cargo delivered by EVs or account for the entirety of the biomolecular repertoire contained therein. Because their cargo may have pleiotropic or opposing actions, intact and bioavailable EVs should be used to evaluate the effects of EVs in biological systems. This naturally raises questions about the best dose of EVs that should be used in experimental modeling. This is important because increasing doses of EVs until an effect occurs treats EVs as “deus ex machina”. Specifically, when EVs are introduced at progressively higher doses until there is a robust response, this could provide an artificial answer to the research question. We used a different approach, in which we rationalized that biologically relevant doses would provide better modeling of the effects of EVs as compared to using high concentrations of EVs that are not likely to be physiologically relevant but could provide a biological response.

C2C12 cells are an immortalized mouse myoblast cell line that differentiate into myotubes when grown to confluence and then differentiate in serum‐containing media. The serum in the media provides a source of circulating EVs for the differentiating cells, which enhances myogenic function (Aswad et al., 2016). In the current study, we matched the circulating EV content from a murine model of OT2D in cell culture media that contains no EVs with the EV content of standard differentiation media incubated on mature myotubes.

The purpose of this study was to evaluate the effects of circulating EV's isolated from plasma of control and OT2D mice on C2C12 myotubes using biologically relevant doses of EVs (i.e., doses equivalent to circulating EVs cells that would be generated in normal culture conditions) to identify signaling differences associated with OT2D. We hypothesized that circulating EV's from OT2D mice would alter cell signaling and the metabolic phenotype of C2C12 myotubes in a manner that mirrors the OT2D condition. Because microRNAs are a common cargo of circulating EVs (Xu et al., 2022) that regulate gene expression via targeting specific mRNAs (Cannell et al., 2008), this work will use gene expression as a primary outcome measure.

## 2 MATERIALS AND METHODS

### 2.1 C2C12 cell culture

C2C12 myoblasts were purchased from ATCC (CAT# CRL‐1772) and maintained in growth media (GM) comprised of Dulbecco's modified eagle medium (DMEM) (Gibco Ref. #11995‐065) supplemented with 10% fetal bovine serum (FBS) (ThermoFisher Ref. #16000044) and 1% antibiotic/antimycotic solution (Gibco Ref. #15240‐062). Myoblasts were passaged before reaching ≈60% confluence until seeding in Matrigel (Corning Ref. #356237)‐coated dishes for experimental use. The plates were coated in 3.5% Matrigel in complete GM for 2 h and washed once with sterile PBS before the cells were seeded. When myoblasts were ≈70% confluent, the GM was replaced with differentiation media (DM), consisting of DMEM supplemented with 2% horse serum and 1% antibiotic/antimycotic solution. Exosome‐free DM was made by supplementing DMEM with 1% exosome‐depleted FBS (ThermoFisher Ref. #A2720803) and a 1% antibiotic/antimycotic solution. This is an alternative formulation for DM that has been used by other labs (Han et al., 2014; Park et al., 2007; Pietrabissa et al., 2010). We utilized this formulation to guarantee exosome depletion in our DM; this is important because exosomes are considered the most bioactive class of EVs. When myotubes had differentiated for 3 days, they were treated with 10 μM arabinosylcytosine (AraC) (Millipore Sigma Ref. #C6645) for 24 h to remove proliferating myoblasts. Myotubes were allowed to differentiate for 5 days in standard DM, at which point they were switched to EV‐free DM. Animal plasma‐derived EVs were added to the EV‐free DM at a dose of 50 μg/mL, which mirrors the dose of EVs found in standard DM.

### 2.2 Animals

All experiments were performed in strict compliance with approved protocols by the UTHSC institutional animal care and use committee (IACUC). Animals were housed in a temperature‐ and humidity‐controlled vivarium with a 12‐h light and dark cycle and given ad libitum access to standard rodent chow (Inotiv Ref. #7912) and water. These experiments utilized the Db/Db mouse model (Jackson Labs Bar Harbor, Maine, Strain #000697), which becomes hyperphagic due to homozygosity for a defective leptin receptor. Control animals were littermates of homozygote Db/Db animals that were heterozygotes for the leptin receptor mutation and do not develop hyperphagia, obesity, or diabetes. Control animals were weaned into the same cages as Db/Db littermates and are therefore considered cage controls. Db/Db and cage control animals had diabetes (or lack thereof) verified using an alphatrak 2 veterinary glucometer (Zoetis). EVs were isolated from the plasma of young (10–16‐week‐old) male Db/Db or cage control animals, EVs were pooled from isolations of at least two animals to minimize the variation associated with using EVs from a single animal and used for downstream analysis of effects on C2C12 myotubes. Western blot preparations and size characterizations of EVs were performed on aliquots of EVs isolated from single animals.

### 2.3 EV isolation

EV's were isolated using ExoQuick isolation reagent (System Bioscience; SBI, Ref. #ExoQ2A‐1) according to the manufacturer's instructions. For isolation from media, 1 mL of ExoQuick was added to 5 mL of media and allowed to incubate overnight before being centrifuged at 3000× *g* for 30 min to precipitate EVs. Media was aspirated, and the EV pellet was lysed in 50 μL of radioimmunoprecipitation assay (RIPA) buffer (ThermoFisher Ref. #89900), and protein was quantified using the Bio‐Rad DC protein assay (Bio‐Rad Ref. #5000111). Protein quantity in the EV pellet was used to determine EV concentration in C2C12 differentiation media. For EV isolations from plasma, 500 μL of whole blood was collected from Db/Db or cage control mice into EDTA‐coated tubes (Sarstedt Ref. #20.1341.102) and centrifuged for 15 min at 3000× *g* to separate the hematocrit. The plasma was placed into a clean microcentrifuge tube and centrifuged for 10 min at 15,000× *g* to remove any circulating cells. Plasma was diluted 1:1 with sterile phosphate buffered saline (PBS) (Corning Ref. #21‐031‐CV) and filtered through a syringe‐mounted 0.2 μm Whatman filter (Cytiva Ref. #6780‐2502). Samples were then incubated for 30 min a 4°C with ExoQuick reagent at a ratio of 200 μL of PBS/plasma mixture to 67 μL of ExoQuick reagent. Samples were then centrifuged for 15 min at 3000× *g*, the supernatant was aspirated, and the resultant EV pellet was resuspended in sterile PBS. Total EV protein was quantified using the Bradford method (ThermoFisher Ref. #23236) so that the EV dose was equivalent across treatments.

### 2.4 EV size characterization

An aliquot of isolated EV's was resuspended in 1 mL of sterile PBS and transferred to an unused BrandTech UV‐transparent disposable cuvette (BrandTech Item # UX‐39458‐60). EVs were then characterized using a ZetaSizer instrument (Malern Panalytical). Measurements were aggregated from three separate sets of 15 measurements. Sample quality analysis was also performed on ZetaSizer software to determine the likelihood of contamination with particles larger or smaller than EVs.

### 2.5 EV labeling

Isolated EVs were labeled with ExoGlow membrane labeling reagent (SBI Inc. CAT# EXOGM600A‐1) according to the manufacturer's instructions. Briefly, EVs were resuspended in sterile PBS, and protein was quantified using a Bradford assay (ThermoFisher Ref. #23236); 100 μg of EV protein was then added to a mixture of 12 μL of reaction buffer and 2 μL of labeling dye. EVs were labeled for 30 min at room temperature, and then EVs were reprecipitated using ExoQuick as described above. Labeled EVs were then incubated on C2C12 myotubes at a concentration of 50 μg/mL overnight. Phase contrast images and images in the excitation/emission spectra of ExoGlow (465 and 635 nm, respectively) after washing twice with sterile PBS to remove EVs not interacting with myotubes or ECM.

### 2.6 Nanoparticle tracking analysis

Nanoparticle tracking analysis (NTA) was performed to validate EV shape, as well as elucidate their concentration, and provide an orthogonal measure of EV size. Briefly, isolated EVs were resuspended in sterile filtered PBS and passed through a 0.2 μm filter. Analysis was then performed on a Nanosight LM10 NTA instrument (Malvern Panalytical) calibrated to 100 nm latex calibration beads. Videos were generated from all biological replicates in this experiment, as well as size characterization and particulate concentration measurements.

### 2.7 2‐NBDG assay

C2C12 myotubes were allowed to differentiate for 5 days, at which point DM was replaced with EV‐free DM supplemented with plasma‐derived EVs from Db/Db or cage control mice. Myotubes were exposed to plasma‐derived EVs for 48 h, then the media was changed to fresh plasma‐derived EV‐containing media at 24 h. After the EV incubation period, the myotubes were incubated overnight in EV‐supplemented 2‐NBDG fasting media (DMEM + 1 g/L glucose +0.25% bovine serum albumin) as described by Bala et al. (2021). The following day, myotubes were incubated in glucose‐free DMEM supplemented with 60 μM 2‐(N‐(7‐Nitrobenz‐2‐oxa‐1,3‐diazol‐4‐yl)Amino)‐2‐Deoxyglucose (2‐NBDG) (ThermoFisher Ref. #N13195) for 2 h with or without the addition of 100 nM insulin. Myotubes were washed three times in sterile PBS to remove any extracellular 2‐NBDG, and then cells were lysed in RIPA buffer. Protein concentration and volume were normalized across all samples, and 2‐NBDG was quantified fluorescently on a Biotek Synergy H2 plate reader (Agilent Technologies) using the excitation/emission spectra of 2‐NBDG (467 and 538 nm, respectively).

### 2.8 Oil Red O staining

Myotubes that had differentiated for 5 days were incubated in EV‐free DM supplemented with plasma‐derived EVs from Db/Db or cage control mice for 24 h. Cells were washed twice in sterile PBS and then fixed in formalin for 10 min at room temperature. The formalin was changed, and the cells were incubated overnight at 4°C. The next day, formalin was removed, and cells were washed twice in distilled water and then once with 60% isopropyl alcohol for 5 min at room temperature. Alcohol was removed, and then the cells were dried completely with a hair dryer. After drying, the cells were stained for 10 min with a 60% solution of 35% Oil Red O in isopropyl alcohol that had been filtered using a 0.2 μm Whatman Filter (Cytiva Ref. #6780‐2502). The Oil Red O was removed, and the myotubes were washed four times with distilled water. Cells were dried, and images were acquired for qualitative analysis on a Biotek Lionheart microscope (Agilent Technologies). Oil Red O accumulation was quantified by eluting in 100% isopropyl alcohol and measuring optical density at 500 nm compared to a 100% isopropyl alcohol blank.

### 2.9 Quantitative real‐time PCR

After 5 days of differentiation in standard DM, C2C12 myotubes were incubated in EV‐free DM supplemented with plasma‐derived EVs for 24 h. RNA was isolated from myotubes using Trizol (ThermoFisher Ref. #15596026) and the PureLink RNA Mini Kit (ThermoFisher Ref. #12183025). RNA was quantified, and the 260/280 ratio was used to verify the purity of the isolated RNA using a Biotek Take3 plate (Biotek Ref. #TAKE3‐SN). RNA samples were treated with DNAse 1 to remove any potential genomic DNA (ThermoFisher Ref. #18068015), and 1 μg of RNA was used for cDNA synthesis using the Roche Transcriptor first‐strand cDNA synthesis kit (Roche Ref. #04897030001). Quantitative real‐time PCR was used to measure gene expression in 384‐well plates using a Roche LC480 qPCR machine and Roche SYBR green PCR master mix (Roche Ref. #04707516001), and custom‐designed primers for genes of interest were obtained from Millipore Sigma custom DNA oligo services (primer sequences are listed in Table S1) or pre‐validated primers from Integrated DNA Technologies (IDT) (sequences are not provided by IDT, however catalog numbers are provided in Table S1). An analysis of common housekeeping genes for skeletal muscle cells was performed, and hypoxanthine phosphoribosyltransferase 1 (HPRT) was selected as the housekeeping gene for this analysis based on consistency within and between groups. Data were expressed as normalized fold change using the 2^−ΔΔct^ method ± standard deviation of the mean. Primer sequences can be found in Table S1.

### 2.10 Western blot

Protein was isolated from both EV preparations and EV‐treated myotubes using RIPA buffer (ThermoFisher Ref. #89901). Precipitated EVs were resuspended in ice‐cold RIPA buffer before being vortexed vigorously for 15 s. Myotubes were serum starved in DM but treated with plasma‐derived EVs overnight before being stimulated with 100 nm insulin for 15 min. All media was removed, and myotubes were washed twice in sterile PBS before RIPA buffer was added directly to wells and cells were collected with a rubber cell culture scraper (Fisher Scientific Ref. #08‐100‐241). All samples were incubated in RIPA buffer on ice for 15 min before being centrifuged for 15 min at 14,000× *g* in a pre‐cooled centrifuge at 4°C. Protein was quantified using the Bio‐Rad DC protein assay (Bio‐Rad Ref. #5000111), and protein concentration was normalized. Western blot samples were prepared from normalized protein using NuPage sample buffer and reducing agent (Invitrogen Ref. #NP0007 and NP0009, respectively) according to manufacturer specifications. Myotube lysate that was not stimulated with insulin was used as a negative control for EV biomarkers (Yoshioka et al., 2013) while AKT phosphorylation (van der Mijn et al., 2014) and calnexin were used as a positive control markers for EVs (Bonsergent et al., 2021). Gel electrophoresis was performed on Western blot samples in NuPage 4%–12% SDS gels (Invitrogen Ref. #NP0336), and proteins were transferred onto pre‐measured nitrocellulose membranes (Bio‐Rad Ref. #1620215). Ponceau staining was performed for 10 min at room temperature to visually confirm the transfer of the proteins to the membrane and to normalize the antibody signal to the total protein loaded on the membrane. Ponceau was removed by three washes for 5 min each at room temperature in tris‐buffered saline containing 1% Tween‐20 (TBST). Blocking was performed for 1 h at room temperature using 5% bovine serum albumin dissolved in TBST. Primary antibodies were incubated overnight at 4°C at a 1:1000 dilution in TBST. Primary antibodies were decanted, and membranes were washed three times for 5 min each in TBST at room temperature. Secondary antibodies were incubated for 1 h at room temperature at a 1:10,000 dilution. Membranes were washed three times for 5 min each in TBST, and then membranes were covered in SuperSignal™ West Pico PLUS Chemiluminescent Substrate (ThermoFisher Ref. #34577) and allowed to incubate for 5 min before imaging on an iBright FL1500 Western blot imaging system (ThermoFisher). Densitometry analysis was performed using the iBright analysis software, where applicable. The antibody catalog numbers are listed in Table S2. When phosphorylation of AKT was measured, all western blot analyses were performed on the same membrane; antibodies were stripped between analyses using Restore Plus western blot stripping buffer (ThermoFisher Ref. #46430).

### 2.11 Statistical analysis

All data are presented as mean ± standard deviation. All comparisons were performed using an unpaired, two‐tailed *T*‐test with Welch's correction, with the exception of 2‐NBDG measurements. An analysis of 2‐NBDG uptake was performed using a 2 × 2 ANOVA for the effects of insulin and EV treatment. Bonferroni post‐hoc analysis was used once the main effect was determined; relevant *p*‐values are listed in the figure. The statistical significance was determined to be *p* < 0.05 for all comparisons. All instances of statistical significance and a strong tendency (*p* < 0.1) have the *p* value for their statistical test listed on the figure. Statistical analyses and figure generation were performed on Prism GraphPad software version 9.5.1.

## 3 RESULTS

### 3.1 EV isolate characterization

**FIGURE 1:**
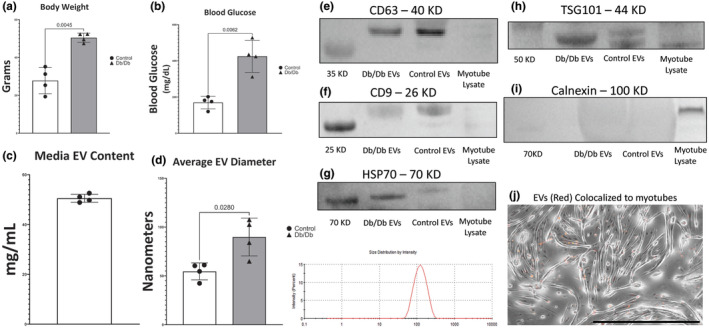
Characterization of a circulating extracellular vesicle (EV) isolate. (a) Body weight of animals immediately before EV isolation. (b) Ad libitum blood glucose of animals immediately before EV isolation. (c) Content of EVs found in standard C2C12 differentiation media. (d) Average EV diameter as calculated by the ZetaSizer instrument, with a representative size trace of EVs isolated from a Db/Db mouse. (e) Western blot image of CD63 compared to myotube lysate as a negative control. (f) Western blot image of CD9 compared to myotube lysate as a negative control. (g) Western blot image of HSP70 compared to myotube lysate as a negative control. (h) Western blot image of TSG101 compared to myotube lysate as a negative control. (i) Western blot image of calnexin in isolated EV preparations compared to myotube lysate‐positive control. (j) EVs are located in extracellular spaces and are also colocalized to myotubes (phase contrast). EVs were identified with ExoGlow membrane labeling (red), and images are shown after incubation overnight with an EV concentration of 50 μg/mL (scale bar in black is 1000 μm).

Every animal from which EVs were isolated in this study was weighed, and blood glucose was measured in an ad libitum state. This confirmed that Db/Db mice were exhibiting an OT2D phenotype and that controls were normoglycemic. Db/Db animals were obese, weighed significantly more (82% increase, *p* = 0.0015; Figure 1a), and had elevated blood glucose (250% increase, *p* = 0.0062) (Figure 1b) relative to controls. For this experimental model, it is important to know the quantity of EVs present in standard differentiation media and to closely appropriate dose EVs on myotubes. We found the mean protein content of EV pellets from media to be 50.6 μg/mL (Figure 1c). We elected to use 50 μg/mL as the dose for supplementing media through all of the experiments.

EV particles are heterogeneous in bioactivity, origin, and size, with a range from 30 nm to 10 μm (Willms et al., 2018). Heterogeneity in the function of EVs of different sizes made it important for us to characterize the particulate size distribution of our isolated EV preps. ZetaSizer analysis comparing circulating EVs from Db/Db mice and cage control mice shows that EVs from diabetic mice have a 64% increase in average diameter compared to controls (*p* = 0.02) (Figure 1d). A representative ZetaSizer analysis graph shows our EV preps contain a monophasic peak in the range of 30–300 nm in diameter (Figure 1d).

Next, our EV isolate was characterized, and its appropriateness for in vitro use was determined. CD63 and CD9 are tetraspanin proteins that are known to be enriched on the surface of EVs (Yoshioka et al., 2013). We analyzed the CD63 and CD9 content in EV isolates compared to lysates of myotubes that were differentiated for 5 days. Qualitative analysis showed clear enrichment of CD63 and CD9 relative to myotube lysate in our EV isolations (Figure 1e,f). HSP70 and TSG101 are proteins that have also been used to demonstrate enrichment of EVs in samples because of protein enrichment in EVs compared to in cell lysates (Xiao et al., 2019). Our EV preps qualitatively show enrichment of HSP70 (Figure 1g) and TSG101 (Figure 1h) relative to myotube lysate.

We next evaluated our protocol for the removal of cellular debris. Calnexin has been used for this purpose in EV isolation previously because calnexin is generally localized to the endoplasmic reticulum of cells and is generally absent from EVs (Mol et al., 2017). Qualitative analysis of our EV prep compared to myotube lysate as a positive control illustrates the exclusion of calnexin from our EV isolates (Figure 1i). This indicates that our EV preps are enriched in known markers of bioactive EVs (Yoshioka et al., 2013) and do not contain measurable amounts of a protein that would show contamination of our preps with cellular debris containing endoplasmic reticulum.

We then confirmed that EVs, as dosed in our model, were able to colocalize with myotubes as well as occupy extracellular space. When ExoGlow‐labeled EVs have been incubated with myotubes in EV‐free DM at this dose, imaging shows that EVs (in orange) effectively colocalize to myotubes (shown in phase contrast) after PBS washing (Figure 1h). A portion of the EVs, however, remain outside of myotubes; this is expected and necessary for complete EV‐mediated signaling. This indicates that our isolated EVs are intact and bioavailable for in vitro use.

### 3.2 Nanoparticle tracking analysis (NTA)

**FIGURE 2:**
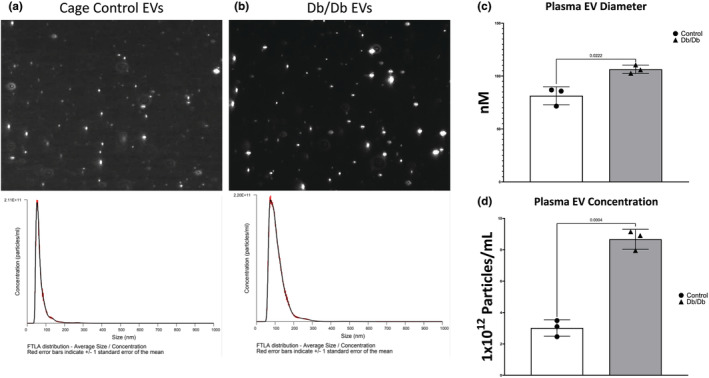
Nanoparticle tracking analysis of plasma extracellular vesicles. (a) Representative plasma EV video frame and size trace from a control mouse. (b) Representative plasma EV video frame and size trace from a Db/Db mouse. (c) Plasma EV diameter as calculated from nanoparticle tracking analysis. (d) Plasma EV concentration as calculated from nanoparticle tracking analysis.

NTA was used to determine that our EVs were the appropriate shape and size, as well as to determine the plasma concentration of EVs isolated from control and Db/Db mice. Representative still photos from NTA videos and size traces corresponding to those analyses show that our EV isolates contain appropriately shaped and sized particles (Figure 2a,b). Similar to our measurements on the ZetaSizer, NTA analysis shows that serum EVs from Db/Db mice are significantly larger than controls (30.5% increase, *p* = 0.022; Figure 2c). Db/Db animals also exhibited a significant increase in the concentration of plasma‐derived EVs relative to controls (187% increase, *p* = 0.0004; Figure 2d). This indicates that our isolation methods yield particles resembling what would be expected for an EV isolate and that the presumably EV particles are circulating in plasma at higher abundance in Db/Db mice.

### 3.3 Glucose uptake

**FIGURE 3:**
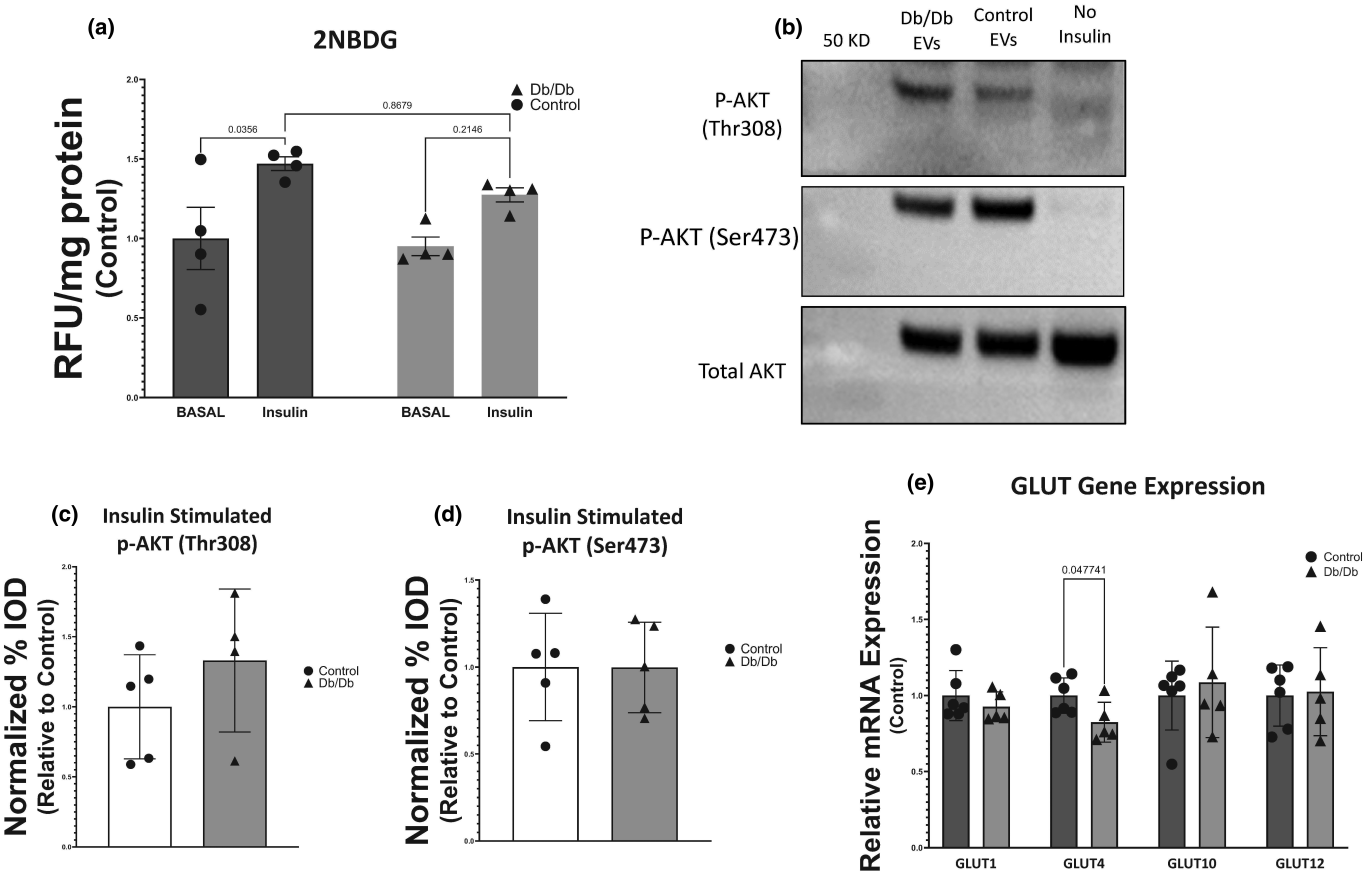
Effect of circulating extracellular vesicles (EVs) on glucose uptake, insulin signaling, and glucose transporter expression. (a) 2‐NBDG uptake in the basal and insulin‐stimulated states of myotubes exposed to plasma‐derived EVs from control or Db/Db animals. (b) Representative images of Western blots for phosphorylated AKT on Thr308 and Ser473 as well as total AKT. (c) Insulin‐stimulated phosphorylation ratio of P‐AKT (Thr308) to total AKT after exposure to circulating EVs. (d) Insulin‐stimulated phosphorylation ratio of P‐AKT (Ser473) to total AKT after exposure to circulating EVs. (e) Relative mRNA quantification genes encoding glucose transporter proteins after exposure to circulating EVs.

The 2‐NBDG uptake assay was used as a proxy measurement for basal and insulin‐stimulated glucose uptake in EV‐exposed myotubes. We analyzed 2‐NBDG uptake using a 2 × 2 ANOVA to determine the effects of insulin and EV treatment. We found that insulin significantly increased 2‐NBDG uptake in control EV‐treated myotubes (47% increase, *p* = 0.0356) but Db/Db EV‐treated myotubes did not exhibit a statistically significant increase in glucose uptake (Figure 3a). Surprisingly, the decrease in insulin‐stimulated 2‐NBDG uptake did not coincide with a decrease in AKT2 phosphorylation on either Thr308 (a phosphorylation site canonically activated by PDK1) or Ser473 (a phosphorylation site canonically activated by mTORC2) (Figure 3b–d).

Quantitative real‐time PCR was used to measure the expression of GLUT genes in myotubes after exposure to circulating EVs from Db/Db or control mice. GLUT1, GLUT4, GLUT10, and GLUT12 mRNAs were all measured. The only significant difference observed in these measurements was GLUT4, which showed a 17% decrease in relative mRNA abundance (*p* = 0.047; Figure 3e).

### 3.4 Regulation of myosin heavy chain expression and muscle phenotype

**FIGURE 4:**
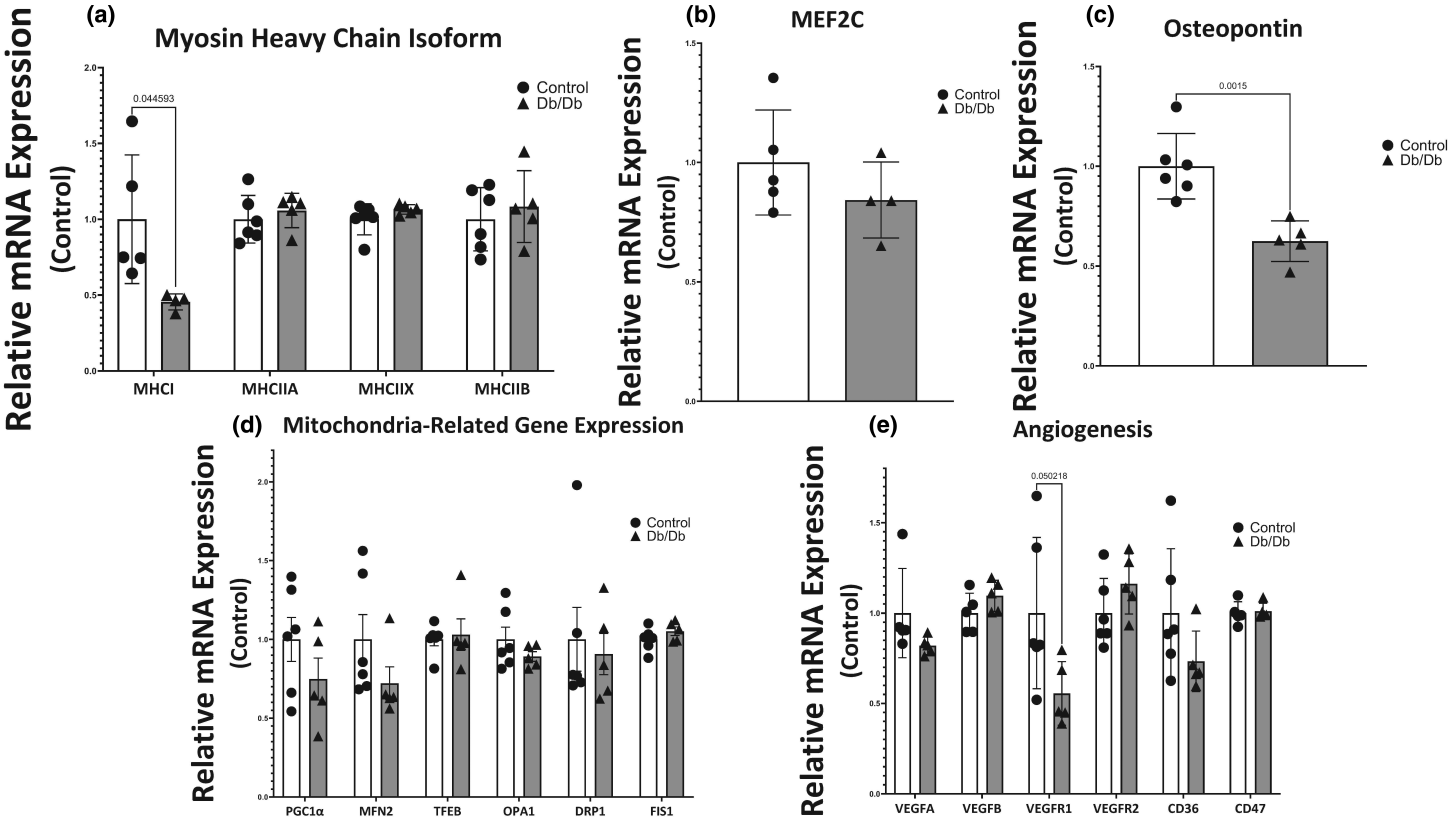
Effect of circulating extracellular vesicles (EVs) on myosin heavy chain isoforms and other determinants of muscle phenotype. (a) Relative mRNA quantification genes encoding myosin heavy chain isoforms after exposure to circulating EVs were isolated from control or Db/Db mice. (b) Relative mRNA quantification of MEF2C. (c) Relative mRNA quantification of osteopontin. (d) Relative mRNA quantification genes encoding proteins necessary for mitochondrial biogenesis and dynamics after exposure to circulating EVs. (e) Relative mRNA quantification genes encoding proteins that regulate angiogenesis in skeletal muscle after exposure to circulating EVs.

Myosin heavy chain isoform gene expression was measured using qPCR. The four primary adult myosin heavy chain genes expressed in mouse skeletal muscle were assayed (MHCI, MHCIIA, MHCIIX, and MHCIIB). It was found that MHCI mRNA was reduced by 54% in myotubes that had been exposed to Db/Db EVs relative to controls (*p* = 0.044) (Figure 4a). Interestingly, there was no significant difference in MEF2C mRNA expression (Figure 4b).

Expression of osteopontin mRNA was measured because osteopontin has known roles in macrophage recruitment and regulation of TGFβ signaling (Uaesoontrachoon et al., 2013; Vetrone et al., 2009). Adipose tissue has been shown to increase osteopontin in OT2D; however, our data show that osteopontin mRNA in myotubes exposed to circulating EVs from Db/Db mice was 37% lower than controls (*p* = 0.0015; Figure 4c).

Expression of a selection of genes relevant for mitochondrial biogenesis, mitochondrial fusion, mitophagy, and mitochondrial fission were also measured because mitochondrial network fractionation has been reported in skeletal muscle in the OT2D state (Lin et al., 2018). PGC1α, MFN2, TFEB, OPA1, DRP1, and FIS1 mRNA were all assessed, and no significant differences were found between myotubes treated with circulating EVs from Db/Db mice or controls (Figure 4d).

Maintenance of the microvasculature in muscle is necessary to deliver adequate oxygen supply and support oxidative capacity; skeletal muscle capillary rarefaction is a well‐established phenotype in OT2D (Groen et al., 2014). Measurement of mRNA transcripts was performed for VEGFA, VEGFB, VEGFR1, VEGFR2, CD36, and CD47. These genes were selected for their importance in maintaining the microvascular network in skeletal muscle. While not statistically significant, VEGFR1 showed a strong tendency to be reduced (difference of means, 44%, *p* = 0.0502) in Db/Db circulating EV‐exposed myotubes relative to controls (Figure 4e). No other genes related to microvascular network maintenance showed any differences in expression.

### 3.5 Lipid accumulation

**FIGURE 5:**
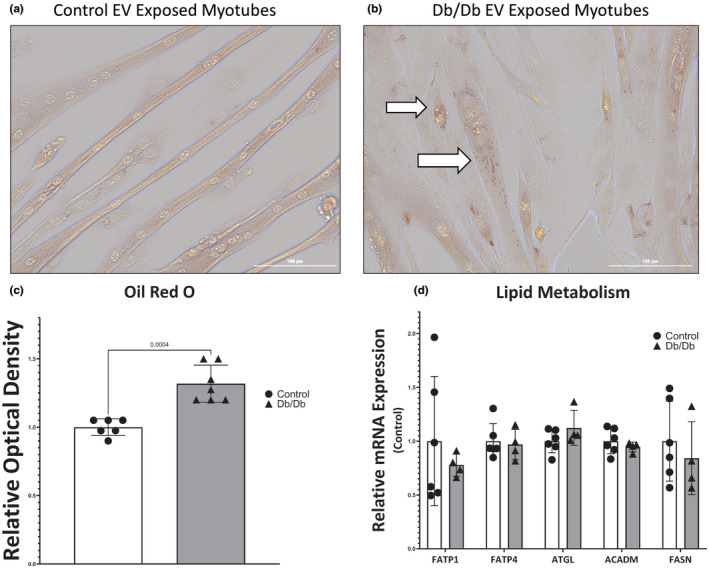
Effect of circulating extracellular vesicles (EVs) isolated from the plasma of control or Db/Db mice on lipid accumulation in myotubes. (a) Representative image of Oil Red O staining in control EV‐treated myotubes. (b) Representative image of Oil Red O staining in Db/Db EV‐treated myotubes. White arrows highlight dense areas of neutral lipid inclusions. (c) Elution of Oil Red O from circulating EV‐exposed myotubes. (d) Relative mRNA quantification genes that regulate lipid entry, lipolysis, and lipid synthesis in skeletal muscle after exposure to circulating EVs.

Oil Red O was used to visualize neutral lipid accumulation in myotubes after exposure to EVs. Qualitative visual inspection shows minimal lipid accumulation in myotubes exposed to control animal circulating EVs (Figure 5a), but a sharp increase in punctate red lipid inclusions in myotubes after treatment with circulating EVs from Db/Db mice (Figure 5b). Elution of Oil Red O was performed after qualitative visual analysis, and consistent with the qualitative appearance, EVs from plasma of Db/Db mice exposed on myotubes, contained 32% more Oil Red O compared to EVs from non‐diabetic control mice (Figure 5c).

To determine if circulating EVs were altering the expression of genes necessary for lipid entry into myotubes, lipolysis, and fatty acid synthesis, qPCR was performed using primers targeting FATP1, FATP4, ATGL, ACADM, and FASN. Surprisingly, none of these genes showed differential expression between Db/Db or control EV‐exposed myotubes (Figure 5d).

## 4 DISCUSSION

In this preliminary study, we examined the effects of circulating EVs from OT2D mice on skeletal muscle myoblasts in culture. Applying EVs in a dose that was consistent with the contents present in standard media allows modeling of the effects of EVs in circulation on C2C12 myotubes in a manner that mimics the contribution of EVs derived from serum in the culture media. While these data model the effects of circulating EVs in OT2D mice on muscle cells, we believe that this model is suitable for a wide range of applications in which systemic EV release or cargo may be impacted.

The characterization of our EV isolate confirms that we used a reasonably pure population of EVs in our study, as evidenced by the enrichment of EV‐associated proteins (Figure 1e–i) as well as orthogonal size characterization by both ZetaSizer and NTA (Figures 1d and 2c). Small EVs are generally described as having diameters of 200 nm and below (Jia et al., 2022). EVs isolated for this study included those with a diameter of up to 300 nm. This indicates that our isolate includes both small and large EVs as they are typically defined. We can report, however, that circulating EVs in this size range are larger on average (Figures 1d and 2c) in Db/Db mice relative to controls. We cannot say definitively that an increase in EV size directly translates to an increase in EV cargo, but increased EV size would logically demonstrate a capacity to carry more or larger biomolecules through the circulation. We also illustrate that EVs isolated from the blood using our protocol remain intact and bioavailable, as evidenced by localization to myotubes after membrane labeling and by video analysis after isolation (Figures 1h and 2a,b).

Our results indicate that in an obese and severely diabetic condition, EVs are circulating in greater abundance than in non‐obese, non‐diabetic controls (Figure 2d). Furthermore, these EVs contribute to skeletal muscle insulin resistance through a mechanism unrelated to AKT2 phosphorylation at either of its canonical phosphorylation sites (Figure 3b–d). Prior work has identified the actions of miRNAs acting on AKT‐interacting proteins as a potential means of EV‐mediated contributions to insulin resistance (Katayama et al., 2019). It is important to note that AKT‐interacting proteins modulate the activation of AKT2 by fostering phosphorylation, indicating that divergent mechanisms appear to be at play in this work and as compared to previous studies; this is however not surprising considering the differences that exist in methodologies and organism models from these studies. While GLUT4 translocation in response to insulin in OT2D is known to be the canonical defect of the disease in skeletal muscle, less is known about the other GLUT family members expressed in skeletal muscle during OT2D. We found a small but statistically significant decrease in GLUT4 mRNA (Figure 3e) in myotubes exposed to circulating EVs from Db/Db mice relative to controls and no other changes in GLUT gene expression. This observation is in opposition to the increases in GLUT4 mRNA and unchanged amounts of protein that have been reported in the literature for decades (Hager et al., 1991). This indicates that circulating EVs likely do not play a significant role in the transcriptional regulation of glucose transporters in diabetic skeletal muscle.

We report that circulating EVs from Db/Db mice specifically reduced the myotube transcript abundance of MHCI mRNA relative to EVs isolated from control mice (Figure 4a). It is known that MHCI expression is not prominent in C2C12 myotubes at this point in differentiation (Brown et al., 2012), but mRNA for MHCI was quantifiable in vitro, and regulation in vivo is not likely to be different. Interestingly, because MEF2C mRNA was not significantly different between experimental groups (Figure 4b), it is likely that the regulation of MHCI is downstream of MEF2C. It is clear from the literature that myosin RNA abundance is often disconnected from translation (Olsson et al., 2011), but specific inhibition of the mRNA pool of one MHC isoform clearly favors a fiber type shift away from that fiber type.

Insulin resistance is also known to cause deficits in skeletal muscle regeneration after injury (Hu et al., 2010). Osteopontin is secreted by multiple cell types, including myocytes and macrophages, and serves as a mediator of inflammatory responses that initiate the skeletal muscle regeneration process (Uaesoontrachoon et al., 2008). Most published studies that have examined the role of osteopontin in OT2D focus on its role in adipocyte and hepatocyte inflammation and report that osteopontin expression is upregulated (Bertola et al., 2009; Zeyda et al., 2011). Less is known about the transcriptional regulation of osteopontin in skeletal muscle in obesity or OT2D; however, our data suggest that circulating EVs from OT2D mice blunt osteopontin transcription (Figure 4c). It is reasonable to speculate based on this data that circulating EVs may contribute to the deficits in skeletal muscle regeneration by targeting osteopontin and blunting macrophage recruitment and satellite cell activation in the affected area.

Genes associated with mitochondrial biogenesis and network dynamics were unchanged in this model (Figure 4d). While the role of circulating EVs in regulating PGC1‐α expression in OT2D is not well described, there is evidence to suggest that hyperglycemia and hyperinsulinemia will suppress PGC1‐α mRNA (Pagel‐langenickel et al., 2008). Suppression of mitochondrial fusion is a plausible mechanism for disruption of the mitochondrial network, but the strongest evidence in the literature supports the notion that mitochondrial fission is upregulated in OT2D. While DRP1 is primarily regulated through phosphorylation (Jahani‐Asl & Slack, 2007), Houzelle et al. report that FIS1 expression is transcriptionally regulated in OT2D (Houzelle et al., 2020). Our data would indicate that FIS1 transcriptional regulation is independent of circulating EVs in OT2D.

Dysregulation of angiogenic signaling in skeletal muscle has been shown to occur in obesity as well as OT2D (Walton et al., 2015; Weiss et al., 2022). Our data showed a strong tendency for VEGFR1 mRNA to be decreased after exposure to circulating EVs from Db/Db mice (Figure 4e). This is consistent with Hazarika et al. (2007), who reported decreased VEGFR1 mRNA in the muscle homogenate of diabetic mice. This suggests a potential role for circulating EVs in regulating the skeletal muscle angiogenic signaling cascade in response to metabolic perturbations.

We found a significant increase in neutral lipid accumulation in myotubes exposed to Db/Db mouse EVs (Figure 5a–c). Intramuscular lipid accumulation has been shown to occur in OT2D (Gilbert, 2021); however, we did not observe any differences in genes related to lipid entry, catabolism, or synthesis (Figure 4d). This suggests that our EV isolate is delivering neutral lipids to myotubes. We cannot at present effectively remove VLDL particles that are known to be within the size range of our EV isolate (Rensen et al., 2001) from our preparation. However, single monophasic peaks in our size distribution with peaks larger than the accepted range of VLDL particulates indicate that they are not a major component of our EV isolate. In line with the hypothesis that EVs deliver lipids to muscle in vivo, Blandin et al. report that lipid content in adipose‐derived EVs is altered in the obese state (Blandin et al., 2023). This includes an enrichment of triacylglycerols in EVs from the adipose tissue of obese mice. Triacylglycerols are neutral lipids that would be bound by Oil red O, as seen in this experiment and we speculate that EVs from adipose tissue could be shuttling lipids to the skeletal muscle in the OT2D phenotype for oxidation.

The focus of this project was to determine if EVs at a biologically relevant concentration would exert meaningful biological effects consistent with the OT2D phenotype; however, we recognize that the study has several limitations. First, we must acknowledge the pitfalls associated with our EV isolation protocol. ExoQuick and other polymer‐based precipitation reagents are regarded as “high yield, low purity” EV isolation methods. This is because the precipitates may be contaminated with cellular debris, protein aggregates, and other potentially bioactive molecules. Our validation of our isolation does not suggest any of the most common contaminants are present; however, it is not possible to test for every possible impurity coming from complex biological mixtures such as plasma. We also matched our EV doses based on total protein, which, in the event of a protein contaminant, may significantly decrease the bioactive fraction of the EVs that we used for incubation on the myotubes. This project shows that EVs can be studied in a “cell culture model”, with concentrations that impact muscle cell metabolism and gene expression. However, this model does not allow us to determine the contributions of different organs to EVs, the stimulus for EV secretion, or the cargo contained within the EVs that may be exerting these effects on muscle cells in culture. Furthermore, this study used only male EV donors, and additional work will be needed to identify if there is a sex difference in the effects of circulating EVs on skeletal muscle in OT2D. Nevertheless, our observations provide evidence that a biologically relevant dose of circulating EVs is given to myotubes, contributes to insulin resistance, and alters gene expression in a manner consistent with the skeletal muscle phenotype observed in OT2D. Future work is ongoing to identify the mechanisms that regulate these EV‐induced changes in insulin resistance.

## AUTHOR CONTRIBUTIONS

The experiments were designed by Christopher R. Pitzer. The experiments were conducted by Christopher R. Pitzer and Hector G. Paez. Discussion and interpretation of the data were conducted by Christopher R. Pitzer, Hector G. Paez, Peter J. Ferrandi, Junaith S. Mohamed, and Stephen E. Alway. Statistical analysis alongside manuscript and figure preparation were conducted by Christopher R. Pitzer. The manuscript was written by Christopher R. Pitzer and edited by Christopher R. Pitzer, Hector G. Paez, Peter J. Ferrandi, Christopher R. Pitzer, Junaith S. Mohamed, and Stephen E. Alway.

## CONFLICT OF INTEREST STATEMENT

The authors declare that there are no conflicts of interest.

## ETHICS STATEMENT

All study procedures were approved by the Institutional Review Board of the University of Tennessee Health Science Center (IACUC# 21‐0282.0), which is accredited by Association for Assessment and Accreditation of Laboratory Animal Care International (AAALAC).

## Supporting information


**TABLE S1.** Primer sequences for genes in the order described in the manuscript; catalog numbers listed for pre‐validated primers purchased from IDT (Integrated DNA Technologies, Coralville, IA).
**TABLE S2.** Name and catalog number of antibodies used in the manuscript. System Biosciences Inc. (SBI, Paolo Alto, CA). Cell Signaling Technology (Cell Signaling, Danvers, MA).

## Data Availability

The datasets generated during and analyzed during the current study are available from the corresponding author upon reasonable request.
