# Supplementary material for: Extracellular vesicles from obese and diabetic mouse plasma alter C2C12 myotube glucose uptake and gene expression
Source: Physiol Rep. 2024 Jan 2;12(1):e15898. doi: 10.14814/phy2.15898 (PMC10761623; doi:10.14814/phy2.15898)
Supplement: Supplementary file 1 — TABLE S1. Primer sequences for genes in the order described in the manuscript; catalog numbers listed for pre‐validated primers purchased from IDT (Integrated DNA Technologies, Coralville, IA). TABLE S2. Name and catalog number of antibodies used in the manuscript. System Biosciences Inc. (SBI, Paolo Alto, CA). Cell Signaling Technology (Cell Signaling, Danvers, MA). [file PHY2-12-e15898-s001.pdf]

SUPPLEMENTAL TABLES

Supplemental Table 1

Gene Name	Primer Sequences
HPRT	Forward AGGGATTTGAATCACGTTTG<br>Reverse TTTACTGGCAACATCAACAG
GLUT1	Forward AGTGACGATCTGAGCTACGGG<br>Reverse TCCTCCCACAGCCAACATGAG
GLUT4	Forward TTCACGTTGGTCTCGGTGCT<br>Reverse TAGCTCATGGCTGGAACCCG
GLUT10	Forward TGGTCTACGTGAGCGCCTTC<br>Reverse AGAGCAGGAAGGTCCAAGCC
GLUT12	Forward TGTCAGCGCGGTAGGTC<br>Reverse AGGAACGTAAACATGCTGGGAC
MHCI	Forward TGGACCCACGGTCGAAGTTG<br>Reverse CCATCTCGGCGTCGGAACTC
MHCIIA	Forward AAGCGAAGAGTAAGGCTGTC<br>Reverse CTTGCAAAGGAACTTGGGCTC
MHCIIB	Forward GAAGAGCCGAGAGGTTCACAC<br>Reverse CAGGACAGTGACAAAGAACGTC
MHCIIX	Forward GAAGAGTGATTGATCCAAGTG<br>Reverse TATCTCCCAAAGTTATGAGTACA
MEF2C	Forward GATGAAGAAGGCTTATGAGCTGAGCGTGCTGTGCGACTGTGAG<br>Reverse CTGTTATGGCTGGACACTGGGATGGTAACTGGCATCTCAAAG
PGC1α	Forward TCGCTCAATAGTCTTGTTCTCAA<br>Reverse AGAAGTCCCATACACAACCG
MFN2	Forward CCATGTGTCGCTTATCCTTCT<br>Reverse CATCTTTCTGACTCCAGCCAT
TFEB	Purchased from IDT<br>CAT# Mm.PT.58.7141096
OPA1	Purchased from IDT<br>CAT# Mm.PT.58.7956692
DRP1	Forward GCAACTGGAGAGGAATGCTG<br>Reverse CACAATCTCGCTGTTCTCGG
FIS1	Purchased from IDT<br>CAT# Mm.PT.56a.21878911
VEGFA	Forward CTGCTGTAACGATGAAGCCCTG<br>Reverse GCTGTAGGAAGCTCATCTCTCC
VEGFB	Forward TGTTCCGGGCTGGGACTCTA<br>Reverse GCTTGTCACTTTCGCGGCTT
VEGFR1	Forward GCATCCCTCGGCCAACAATC<br>Reverse TCAGCCACCACCAATGTGCTA
VEGFR2	Forward GATCCCAGATGACAGCCAGACAG<br>Reverse AAAGTCCTGCCTCGTCGCTG
CD36	Forward TGAATGGTTGAGACCCCGTGC<br>Reverse CCATCCACCAGTTGCTCCACA
CD47	Forward GGTGGGAAACTACACTTGCGAAG<br>Reverse CTCCTCGTAAGAACAGGCTGATC
Osteopontin	Forward GACCATGAGATTGGCAGTGA

	Reverse GGAACTGTGTTTTTGCCTCTT
FATP1	Forward GATCAGGCAAGCTCCAGCACAG<br>Reverse GGTCCACGGAAGTCCCAGAAA
FATP4	Forward CCAGTGCTTACTCCACGGCA<br>Reverse AAACGGCTGGAGAAGTCGGT
ATGL	Forward ACCTTCGCAATCTCTACCGCC<br>Reverse AGCAAAGGGTTGGGTTGGTT
ACADM	Forward GAGGATGACGGAGCAGCCAA<br>Reverse GGGTGTCGGCTTCCACAATG
FASN	Forward CCGTGAGTCTATCCTGCGCT<br>Reverse ACAGGCAACCTCTCCCAAGG

Supplemental Table 1: Primer sequences for genes in the order described in the manuscript, Catalog numbers listed for pre-validated primers purchased from IDT (Integrated DNA technologies, Coralville, IA).

Supplemental Table 2

Antibody Name	Company Catalog #
CD81	SBI #EXOAB-CD81A-1
Calnexin	Cell Signaling #2433
P-AKT (Thr308)	Cell Signaling #9275
P-AKT (Ser473)	Cell Signaling #9271
Total AKT	Cell Signaling #9272
Anti-Rabbit IgG Secondary (HRP Conjugate)	Cell Signaling #7074

Supplemental Table 2: Name and catalog number of antibodies used in the manuscript. System Biosciences Inc. (SBI, Paolo Alto, CA). Cell Signaling Technology (Cell Signaling, Danvers, MA).
